# Decidual stromal cells-derived exosomes incurred insufficient migration and invasion of trophoblast by disturbing of β-TrCP-mediated snail ubiquitination and degradation in unexplained recurrent spontaneous abortion

**DOI:** 10.1186/s40001-023-01598-2

**Published:** 2024-01-09

**Authors:** Miao Xiong, Qiaohong Wang, Xiaoxin Zhang, Liping Wen, Aimin Zhao

**Affiliations:** 1 Department of Obstetrics and Gynecology, Renji Hospital, School of Medicine, Shanghai Jiao Tong University, 160 Pu Jian Road, Shanghai, 200127 People’s Republic of China; 2 Shanghai Key Laboratory of Gynecologic Oncology, Shanghai, People’s Republic of China; 3 Department of Obstetrics and Gynecology, Shanghai Jiao Tong University Affiliated Sixth People’s Hospital, Shanghai, People’s Republic of China

**Keywords:** Decidual stromal cells, β-Transducin repeat containing protein, Epithelial–mesenchymal transition, Exosome, Transcription factor snail

## Abstract

**Background:**

Exosomes released from decidual stromal cells (DSC-exos) play a crucial role in facilitating the epithelial–mesenchymal transition (EMT) of trophoblasts and insufficient trophoblasts EMT are associated with URSA (unexplained recurrent spontaneous abortion). However, the mechanisms underlying DSC-exos inducing EMT is not completely understood.

**Methods:**

DSC-exos of normal pregnant women (N-DSC-exos) and URSA patients (URSA-DSC-exos) were extracted and characterized. Characterization of the isolated DSC-exos was performed using with TEM (transmission electron microscopy), NTA (nanoparticle tracking analysis), and WB (western blot) techniques. Subsequently, these DSC-exos were co-cultured with trophoblasts cell lines (HTR-8/SVneo). The influence of both N-DSC-exos and URSA-DSC-exos on trophoblasts proliferation, invasion and migration, as well as on the expression of EMT-related proteins, was evaluated through a series of assays including CCK8 assays, wound healing assays, transwell assays, and western blot, respectively. Then rescue experiments were performed by β-TrCP knockdown or β-TrCP overexpressing trophoblasts with snail-siRNA transfection or β-TrCP overexpressing Lentivirus infection, respectively. Finally, animal experiments were employed to explore the effect of N-DSC-exos on embryo absorption in mice.

**Results:**

We found increased β-TrCP expression in the villus of URSA patients when compared to the normal pregnant women, alongside reduction in the levels of both snail and N-cadherin within URSA patients. N-DSC-exos can promote the EMT of the trophoblast by inhibiting β-TrCP-mediated ubiquitination and degradation of transcription factor snail. Moreover the capacity to promote EMT was found to be more potent in N-DSC-exos than URSA-DSC-exos. Down-regulation of snail or overexpression of β-TrCP can reverse the effects of N-DSC-exos on trophoblast. Finally, in vivo experiment suggested that N-DSC-exos significantly reduced the embryo resorption rate of spontaneous abortion mouse model.

**Conclusions:**

Our findings indicate that URSA-DSC-exos caused insufficient migration and invasion of trophoblast because of disturbing of β-TrCP-mediated ubiquitination and degradation of EMT transcription factor snail. Elucidating the underlying mechanism of this dysregulation may shed light on the novel pathways through which DSC-exos influence trophoblast function, thereby contributing to our understanding of their role in URSA.

**Supplementary Information:**

The online version contains supplementary material available at 10.1186/s40001-023-01598-2.

## Background

Pregnancy is a complex process associated with numerous biological changes in the maternal body and our understanding of the complicated relationship between the mother and its semi-allograft fetus is still limited. Implantation is the key step of pregnancy establishment, in which embryos are demanded to adhere to receptive endometrium and invade the latter. Successful implantation depends on the appropriate and coordinate state of both the maternal and the embryo. Trophoblast play an essential role in establishing early pregnancy and only when trophoblast invasion into the uterus decidua is accomplished can a successful pregnancy be established [1–3]. Placenta extravillous cytotrophoblast invasion involves a cellular transition from epithelial to mesenchymal phenotype [4–6]. The EMT is defined by which polarized epithelial cells lose polarity and intercellular contraction and acquire mesenchymal cell motility [5, 6], and is critical for the implantation of trophoblast. Previous studies have indicated that insufficient migration and invasion of EVT (extravillous trophoblasts) can lead to disrupted maternal–fetal crosstalk, a phenomenon implicated in RSA (recurrent spontaneous abortion) [7–9]. However, the underlying biology or mechanism is largely unknown and thus is the focus of this study. A better understanding of molecular pathways driven by placental EMT, along with the identification of related signaling pathways, could pave the way for innovative therapeutic approaches. These approaches may improve fetal–placental development and address complications such as PE (preeclampsia), RSA and FGR (fetal growth restriction).

Exosomes are diminutive extracellular vesicles, with a size range of 30–150 nm, that are integral to cell-to-cell communication, which carrying various bioactive proteins, mRNAs, miRNAs, and lipids [10]. These nano-scale vesicles are released into the extracellular fluid and have received an emerging interest because it has been believed that they have specialized function such as stimulating immune system and play a key role in many biological events including coagulation as well as intracellular communication [11, 12]. Effects of exosomes on target cells depend not only on their concentration but also on their intrinsic components. The role of exosomes-mediated communication between the maternal endometrium and the conceptus has drawn much attention. Recent studies indicated that exosomes are synthesized and secreted by various components of the female reproductive tract, such as oviductal epithelium, follicular fluid, endometrium, uterine environment, embryos in culture media, and the placenta [13–23] which are necessary for the normal functioning of the placenta and fetal development. Endometrially derived EVs (extracellular vesicles) have been detected in uterine fluid during the critical period for embryo implantation. It has been observed that EVs associated with recurrent implantation failure (RIF-EVs) can adversely affect embryonic development by hindering blastocyst formation, reducing the total cell count in embryos, and impairing the embryos' ability to invade the endometrium [24, 25]. However, the distinctions in the molecular cargo of RIF-EVs compared to those from fertile women (FER-EVs) remain to be fully elucidated. Furthermore, the role of DSC-exos in the process of embryo implantation into the mother are not well understood, underscoring the need for further research to clarify the direct link between DSC-exos and successful embryo implantation.

The study hypothesized that DSC-exos of patients with URSA may exert distinct effects on implantation processes when compared to those from healthy pregnant controls. We procured DSC-exos from both URSA patients and normal pregnant women to establish a co-culture system with trophoblasts. This model was utilized to investigate the modulatory effects of DSC-exos of URSA patients on the implantation potential of trophoblasts.

## Methods and materials

### Study participants

From September 2020 to December 2021 a total of 16 women with URSA, 24 women with clinical NP (normal pregnancy) who underwent negative pressure aspiration surgery at RenJi Hospital were enrolled in the study, in addition, there are 5 non-pregnant women as controls. The clinical charateristics of the participants are listed in Additional file 1: Table S1.

Criteria for inclusion in the URSA group were stringent: (1) exclusion of uterine malformation; (2) fetal heartbeat had ceased or never detected; (3) two or more previous spontaneous abortions; (4) absence of endocrine, metabolic, autoimmune diseases, thrombophilia, or infection; (5) age 22–40 years old, gestational age 6–9 weeks; (6) spouses and individuals with normal chromosomes. Exclusion criteria: all selected embryos undergo chromosomal examination, and any chromosomal abnormalities will be excluded.

We employed normal early pregnancy women undergoing induced abortion for non-medical reasons to terminate pregnancy as a control group. Criteria for inclusion in the normal pregnancy were as follows: (1) the fetal heartbeat of the NP group was verified by ultrasound before elective termination of pregnancy. (2) No history of adverse pregnancy or childbirth in the past. (3) Absence of endocrine, metabolic, autoimmune diseases, thrombophilia, or infection. (4) There were no abnormal symptoms such as vaginal bleeding and abdominal pain during this pregnancy. (5) No history of medication use during pregnancy. (6) Age 22–40 years old, gestational age 6–9 weeks. (7) Spouses and individuals with normal chromosomes. Exclusion criteria: all selected embryos undergo chromosomal examination, and any chromosomal abnormalities will be excluded.

Criteria for inclusion in the non-pregnant women were as follows: (1) perform hysteroscopy for non-pathological reasons and postoperative pathology suggests no pathological changes in the endometrium. (2) No internal, surgical or gynecological diseases. (3) Age 22–40 years old, non-pregnancy. Exclusion criteria: postoperative pathology suggests endometrial lesions.

We performed negative pressure aspiration surgery to obtain decidual tissue samples, ensuring no medication was administered to participants before or during the procedure. The perioperative details of URSA patients are the same as those of the control group. Decidual tissues were collected from gestational week 6–9, immediately post-surgery under aseptic conditions, and washed in cold PBS to remove blood and fetal remnants. Each study participant provided their signed written informed consent for participation, and the study protocol was approved by the Human Research Ethics Committee of the Renji Hospital and written informed consent was obtained from all participants (Ethics approval number: RA-2020-063).

### Isolation, culture and identification of DSCs/ESCs

Specimens of human decidua and endometrium were aseptically collected and promptly transported to the research facility, preserved in chilled 1640 RPMI medium (Gibco, Grand Island, NY, USA). The obtained decidua were pooled, washed in phosphate buffered saline (PBS), and minced into 1–2 mm pieces. The minced tissues were digested by 0.25% trypsin (Gibco, Grand Island, USA) and 0.3% (g/mL) collagenase type II (Solarbio science & technology Co., Ltd, Beijing, China,) in sterile centrifuge tube at 37 °C water bath with gentle shake for 20 min, followed by a repeated cycle of 15 min digestion. Cell suspension was successively filtered through sterile 100-mesh and 200-mesh wire sieves for the DSCs and more 400-mesh wire sieves for the ESCs. The filtered suspension was centrifuged at 1500 rpm for 5 min. After the supernatant was discarded, the cell pellets were resuspended in 1640 RPMI containing 10% fetal bovine serum (FBS) (Gibco, Grand Island, NY, USA), 100 IU/mL penicillin, and 100 mg/mL streptomycin at 37 °C in a humidified environment with 5% CO_2_. After 2 h of culture, primary DSCs adhered to the wall and medium were changed and suspend cells such as leukocytes and erythrocytes were aspirated away. To maintain the decidualization state, DSCs were cultured with a basic medium containing 0.1 μM E2 and 1 μM P4, as described by our previous research [26].

### Identification of DSCs/ESCs by immunofluorescence

In the culture plate, the glass slide which had climbed the DSCs or ESCs were soaked in PBS for 3 times, 3 min each time. Fix the slide with 4% paraformaldehyde for 15 min, and soak the slide with PBS for 3 min each time. 0.5% Triton X-100 (PBS preparation) was permeabilized at room temperature for 20 min. The slides were soaked in PBS for 3 times, each time for 3 min. Then the normal goat serum was dripped onto the slides and sealed at room temperature for 30 min. Discard the sealing fluid and then add enough diluted primary antibody (rabbit anti-human CK-7 antibody and rabbit anti-human vimentin antibody) (Biolegend, California, USA) to each slide and put it into wet box, incubate at 4 °C overnight. The second day: adding fluorescent secondary antibody: PBST was used to soak the slides for 3 times, 3 min each time. After discarding the excess liquid on the slides, diluted fluorescent secondary antibody (Alexa Fluor 488 Affinipure donkey Anti-rabbit IgG) was added (Yeasen biotech, Co., Ltd, Hong Kong). The slides were incubated in a wet box at room temperature for 2h, and PBST was used to soak the slices for 3 times, 3 min each time. For nuclear staining, DAPI was dripped and incubated in dark for 5 min. After removing excess liquid with absorbent paper, the slides were mounted with an anti-fade mounting medium to prevent photobleaching. Finally, the samples were ready for examination and image acquisition using a fluorescence microscope.

### Exosome isolation

Following incubation, the supernatant of DSCs or ESCs were collected from third to sixth cell passage number, centrifuged at 300 g for 10 min, 2000 g for 10 min, and 10,000 g for 30 min to eliminate cells, dead cells, and cell debris. The collected media were then ultracentrifuged at 100,000 g for 70 min. The exosome pellet was washed with PBS, ultracentrifuged at 100,000 g for 70 min, resuspended in 50 μL of PBS and stored at − 80 °C. The protein content was measured using the BCA Protein Assay Kit (Beyotime, China). We obtained approximately 20–30 μg of exosomes from 10 mL of culture supernatant.

### Exosome identification: TEM, NTA, and WB

The exosome samples were retrieved from storage at − 80 °C refrigerator and immediately transferred to an ice-containing container to thaw. After dissolving, the exosome samples were centrifuged slightly. A pipette gun was used to suck 15 µL exosome samples, and the exosome samples were placed on the copper net for 1 min. Filter paper was used to suck up the exosome samples on the copper mesh, and then 15 µL 2% uranyl acetate staining solution was drawn by pipette gun for 1 min at room temperature. Filter paper was used to suck up the exosome samples on the copper mesh, and the dyed samples were roasted under the lamp for 10 min, then observed and photographed by TEM (Tecnai, G2 spirit FEI), with the resulting micrographs being duly recorded for analysis.

The sample cell was cleaned with deionized water, the instrument was calibrated with polystyrene microspheres (110 nm), and the sample cell was cleaned with 1 × PBS buffer (biological industries, Israel). The sample was diluted with 1 × PBS buffer (Bi, Israel) [dilution factor 300 for N-DSC-exos and 100 for URSA-DSC-exos and 40 for ESC-exos]. Following dilution, the sample was loaded into the ZetaView PMX 110 instrument (manufactured by Particle Metrix) for particle analysis and characterization.

The protein concentration of exosomes was determined by using a BCA Kit (Beyotime, Jiangsu, China) according to the manufacturer’s instructions. A 30 µg sample of protein from each extract was separated by 12% SDS-PAGE, and the protein bands were transferred onto polyvinylidene fluoride (PVDF) membranes, the membranes were incubated overnight at 4 °C with primary antibodies against Anti-CD9 antibody, Anti-CD63 antibody, Anti-TSG101 antibody with exosomes and 293 T cells were used as positive controls.

### PKH67-labeled exosomes

Retrieve the exosome and place it on ice until it has thawed. Subsequently, incorporate Diluent C until a final volume of 500 µL is achieved, ensuring thorough mixing to create Solution A. Concurrently, combine 500 µL of Diluent with 4 µL of PKH67 dye in a sterile 1.5 mL Eppendorf tube, mixing thoroughly to produce Solution B. Proceed to mix Solutions A and B, allowing them to incubate at room temperature for a duration of 5 min. To halt the staining process, introduce 1% Bovine Serum Albumin (BSA) for one minute. Subsequently, augment the volume of the mixture to 20 mL using 1% BSA and subject it to ultrahigh-speed centrifugation at 120,000 g for 60 min. Following centrifugation, discard the supernatant and resuspend the PKH67-labeled exosome pellet in sterile PBS.

### Tracing of exosomes

Aspirate the supernatant from the HTR-8/SVneo cell culture and perform three washes with serum-free medium. Subsequently, resuspend the cells in serum-free medium and incubate with 10 µg per well of PKH67-labeled exosomes. Ensure homogeneous distribution of the exosomes by gentle mixing. Incubate the cells at 37 °C with 5% CO_2_ for designated time points of 0, 6, 12, 24, and 48 h. Upon completion of each incubation period, remove the medium and perform three PBS washes. Fix the cells with 4% paraformaldehyde, followed by discarding the fixative and washing thrice with sterile PBS. Permeabilize the cells with 0.1% Triton X-100 in PBS for 5 min, then wash three times with PBS. To block non-specific binding, incubate the cells with a DAPI-containing blocking solution. Examine and document the cellular uptake of exosomes using a laser confocal microscope.

### Cell culture and treatment

The human trophoblast cell line (HTR-8/SVneo) was purchased from Yihe Biotechnology Co., Ltd (Shanghai, China). The HTR-8/SVneo were grown in RPMI-1640 medium (Gibco, Waltham, MA, USA) containing 5% FBS (Gibco, Waltham, MA, USA) in a humidified atmosphere containing 5% CO_2_ at 37 °C, HTR-8/SVneo were either infected with overexpression lentivirus for β-TrCP or transfected with small interfering RNA targeting snail or β-TrCP, and corresponding negative control (si-NC, respectively), all of which were synthesized and purified by Obio Technology (Shanghai, China). ALL transfection procedures were performed using RFect siRNA Transfection Reagent (Baidai biotechnology Co., Ltd, Changzhou, China). HTR-8/SVneo cells were treated with different concentrations of N-DSC-exos, URSA-DSC-exos or ESC-exos before and after siRNA transfection or overexpression lentivirus infection, and then treated HTR-8/SVneo with different concentrations of protease inhibitor MG132 (Yuanye Biotechnology Co., Ltd, Shanghai, China).

### Wound healing assay

HTR-8/SVneo with or without different concentrations of exosomes (5 µg/mL, 10 µg/mL or 20 µg/mL) were seeded into 6-well plates at a density of 1 × 10^5^ per well to ensure that cells reached confluence by the subsequent day. These cells underwent a scratch wound assay where a sterile 10 µL pipette tip was used to create a uniform scratch at 100% confluence. The cell debris were washed away with 1 × PBS, and the remaining cells were cultured with fresh serum-free medium. Wound width was monitored by phase contrast microscopy at regular intervals at 6 h, 12 h, 24 h, 48 h. The rescue experiments were performed with the exosomes concentration 20 µg/mL. The extent of cell migration was assessed by calculating the wound closure, and the obtained images were quantified and evaluated using Image software. Scratch migration rate =  (scratch area in 0 h − scratch area in 48 h)/scratch area in 0 h) × 100%.

### Invasion assay

HTR-8/SVneo with or without different concentrations of exosomes (5 µg/mL, 10 µg/mL or 20 µg/mL) were suspended in free serum medium at a density of 5 × 10^4^ cells/mL. And then the rescue experiments were performed with the exosomes concentration 20 µg/mL. 200 μL cell suspension was placed into the upper chamber (8 μm, Corning, NY, USA) precoated with Corning^®^ Matrigel^®^ Matrix (Corning, NY, USA) diluted with RPMI1640 at 1:8. After that, 600-μL of complete medium with 5% FBS were added into the lower chamber. After incubation for 24 h at 37 °C, cells in the upper chambers were carefully removed. Cells invaded through the membrane were fixed by 4% paraformaldehyde and stained with 0.1% crystal violet. The invaded cells were quantified by examining five random fields on the lower membrane surface at × 200 magnification using an inverted microscope. Images of the invaded cells were captured with a Leica microscope and subsequently evaluated. The amount of invasion were counted by image software. The operating steps are as follows: the number of transmembrane cells is converted from Image Type-8 bit to black and white. Select Edit invert to turn the image background to black, then select Image Adjust Threshold, select B&W to adjust the scroll bar to make the cells as white as possible and the background black. Click Apply. Then click Process Binary Watershed, and finally click Analyze Particles. Count is the number of cells.

### CCK8 assay

100 µL cell suspension of HTR-8/SVneo at a density of 1 × 10^5^ cells/mL was prepared in 96-well plate, and the plate was pre-cultured in incubator for 24 h. Then HTR-8/SVneo cells were incubated with exosomes (20 µg/mL) for 12 h, 24 and 48 h. Following these incubation periods,10 µL CCK8 reagent and 90 µL basic medium were added to each well. After incubation for an additional 1.5 h at 37 °C, the absorbance at 450 nm was measured by microplate.

### Western blot

Total protein was extracted from HTR-8/SVneo cell lines using RIPA lysis buffer (Beyotime, Jiangsu, China), and the protein concentration in each extract was determined by using a BCA Kit from the same manufacturer, adhering to the protocol supplied. For each sample, 20 µg of protein was resolved via 10% SDS-PAGE and subsequently transferred to polyvinylidene fluoride (PVDF) membranes (Beyotime, Jiangsu, China). The membranes were then subsequently blocked using QuickBlock™ Blocking Buffer for (Beyotime, Jiangsu, China)1 h at room temperature. Following this step, the membranes were incubated at 4 °C overnight with primary antibodies against N-cadherin, E-cadherin (from Cell Signaling Technology, Inc, Boston, USA), as well as vimentin, snail, β-TrCP, β-actin (Abcam, Cambridge, USA), and GAPDH (Abcam, Cambridge, USA). The next stage involved incubation with an HRP-labeled secondary antibody for 1 h at room temperature. The protein bands were visualized using ECL-plus reagents (Vazyme Biotech Co., Ltd, Jiangsu, China) with GAPDH or β-actin serving as an internal control.

### Quantitative real-time PCR

Total RNA was extracted from HTR-8/SVneo cell lines using TRIzol reagent (Invitrogen), and cDNA was synthesized by using an Hiscript III RT SuperMix for qPCR (+ gDNA wiper) Kit (Vazyme Biotech Co.,Ltd, Jiangsu, China). The qRT-PCR was performed by using a Cham Universal SYBR qPCR Master Mix Kit (Vazyme Biotech Co.,Ltd, Jiangsu, China) on an ABI 7500 Real-Time PCR System (Applied Biosystems, Foster City, CA, USA). The relative levels of β-TrCP, snail, vimentin, E-cadherin and N-cadherin expression were calculated using the 2^ΔΔCt^ method with GAPDH serving as an internal control. The PCR primer sequences used were as follows: β-TrCP, forward: 5ʹ-ACC AAC ATG GGC ACA TAA ACT C-3′and reverse: 5ʹ-TGG CAT CCA GGT ATG ACA GAA T-3ʹ; GAPDH, forward: 5ʹ-CCCAAAGTCCTCCTGTTTCA-3ʹ, reverse:5ʹ-GTCTTGAGGCCTGAGCTACG-3ʹ. E-cadherin, forward: 5ʹ-GCTCTTCCAGGAACCTCTGTGATG-3ʹ, reverse:5ʹ-AAGCGATGGCGGCATTGTAGG-3ʹ. N-cadherin, forward:5ʹ-AAGGTGGATGAAGATGGCATGGTG-3ʹ, reverse:5ʹ-TGCTGACTCCTTCACTGACTCCTC-3ʹ. vimentin, forward:5ʹ-TTGCCGTTGAAGCTGCTAACTACC-3ʹ, reverse:5ʹ-AATCCTGCTCTCTCCTCGCCTTCC-3’. snail, forward:5ʹ-GGCTCCTTCGTCCTTCTCCTCTAC-3ʹ, reverse:5ʹ-CCAGGCTGAGGTATTCCTTGTTGC-3ʹ.

### Small interfering RNA transfection

The HTR-8/SVneo cell lines were plated with six well plates of antibiotic-free and transfected with siRNA when the cells fused to 30–50%. snail-siRNA, β-Trcp siRNA and NC-siRNA dry powder (Obio Technology, Shanghai, China) was dissolved in sterilized DEPC water. Prepare liquid A and liquid B, liquid A is 250 µL MEM (OPTI-MEM, Gibco, Waltham, MA, USA) + 4 µL β-Trcp siRNA or NC-siRNA, and liquid B is 250 µL MEM + 10 µL transfection reagent RFect siRNA (Baidai biotechnology Co., Ltd, Changzhou, China). After 5 min, mix liquid A and liquid B together. After waiting for 20 min, add the mixed systems of A and B into a six well plate. Before adding the system, the cells need to change the medium (no antibiotics and no FBS). RNA extraction was conducted at the 24 h mark,while protein isolation was performed after 48 h.

### Overexpressing of β-TrCP

Lentiviruses designed to induce β-TrCP overexpression, along with a corresponding negative control, were procured from Shanghai GenePharma in China. The HTR-8/SVneo cell line was plated in 6-well plates and allowed to adhere for 24 h before viral transduction. To facilitate the overexpression of β-TrCP, the cells were treated with polybrene, also supplied by Shanghai GenePharma, upon reaching 40–50% confluence. Subsequent to 48-72 h post-infection, the cells were collected for additional experimental evaluations.

### Co-IP (co-immunoprecipitation)

The detailed protocol for Co-IP is provided in the Supporting Information Methods. Remove media and wash the HTR-8/SVneo cells with PBS. Placed the culture plate on ice, add 1 mL cold Lysis Buffer (Abcam, Cambridge, USA) and keep the plate on ice for one minute. Scraped the cells and gently transferred the disrupted cell suspension into a chilled microcentrifuge tube. Mix on a rotary mixer for 30 min at 4 °C. Centrifuge at 10,000 × g for 10 min at 4 °C and transfer the cell extract to chilled fresh tubes. Add 2.5 µL β-TrCP antibody or snail antibody (Cell Signaling Technology, Boston, USA), along with 2.5 µL IgG (Beyotime, Jiangsu, China) as homotypic control. Adjust the total volume to 500 µL with Lysis Buffer, which containing the Protease Inhibitor Cocktail (Abcam, Cambridge, USA). Prepare the Protein A/G Sepharose (Abcam, Cambridge, USA) by washing 25 µL of the beads twice with 1 mL Wash Buffer, centrifuging at 2000 × g for 2 min and and discarding the supernatant after each wash. After antibody binding, add 25 µL of Protein A/G Sepharose beads slurry to each tube and incubate for 1 h at 4 °C. Collect the Protein A/G Sepharose beads by low speed centrifugation at 4 °C (2000 × g for 2 min), Wash Protein A/G Sepharose beads 3 times with 1 mL 1 × Wash Buffer, collecting the Protein A/G Sepharose beads by low speed centrifugation at 4 °C and aspirating the supernatant in between washes. After the final wash, remove as much of the 1 × Wash Buffer as possible, making sure that the beads never dry completely. To elute the complex, add 40 µL 2 × SDS-PAGE loading buffer to the beads and boil for five minutes. Finally, load the boiled samples onto the wells of SDS-PAGE gel.

### Immunofluorescence

HTR8/SVneo were plated into six-well plates and fixed with 4% paraformaldehyde for 20 min, and then permeabilized with 0.5% Triton X-100 at room temperature for 20 min. For immunofluorescence, the cells were blocked with 5% BSA for 30 min and then incubated with primary antibody against N-cadherin, E-cadherin (Cell Signaling Technology, Inc, Boston, USA), vimentin, snail, β-TrCP (Abcam, Cambridge, USA)at 4 °C overnight. This was followed by the application of fluorescent secondary antibody (Alexa Fluor 488 Affinipure donkey anti-rabbit IgG or Alexa Fluor 596 Affinipure donkey anti-rabbit IgG) (Yeasen biotech, Co., Ltd, Hong Kong) for 1 h in darkness. The nuclei were stained with DAPI (1:100; Sigma-Aldrich) at 37 °C. Positive staining was observed under a fluorescence microscope.

### Immunohistochemistry

The villus of normal pregnant women (24 cases) and URSA patients (16 cases) were collected from 6 to 9 weeks of gestation and fixed in 10% formalin. They were embedded in paraffin blocks that were sliced into sections. The paraffin section (5 μm thickness) were first incubated overnight with primary antibody at 4 °C, including Anti-β-TrCP, Anti-snail (Abcam), Anti-E-cadherin, Anti-N-cadherin, and Anti-vimentin (CST) antibodies were used as the primary antibodies. And this was followed by exposure to the corresponding secondary antibodies at room temperature. Advanced visualization was achieved through the application of an ultrasensitive ABC IgG Staining Kit (Beyotime, China), with diaminobenzidine (DAB) serving as the chromogen for staining. The stained sections were examined under a microscope at 200 × and 400 × magnification to assess the expression of the targeted proteins. The staining results are calculated based on the combination of staining intensity and the percentage of positive cells, and scored separately according to staining intensity and positive rate. Then, the product of staining intensity and positive rate is used as the result of immunohistochemical staining for statistical analysis.

### Animal experiment

44 CBA/J mice, 14 DBA/2 mice and 8 Balb/c mice were employed to explore the effect of DSC-exos on embryo absorption in mice. There were no statistically significant differences in age or weight for the experimental mice we used in this study. We purchased 6–8 week old Balb/c, male DBA/2 and female CBA/J from the Huafukang Biotechnology Co., Ltd (Beijing, China) to establish both the spontaneous abortion and normal pregnancy mouse models. The models utilized were the normal pregnancy mouse model (CBA/J × Balb/c) and spontaneous abortion (CBA/J × DBA/2) phenotypes. The animals were allocated into six distinct groups for the experimental protocols: group1 consisted of normal pregnancy models without any treatment. group2 comprised normal pregnancy models administered with N-DSC-exos. group3, spontaneous abortion mouse model without any treatment. group4 involved spontaneous abortion mouse model treated with PBS. Group5, spontaneous abortion mouse model receiving N-DSC-exos intravenously. Group6, spontaneous abortion mouse model treated with N-DSC-exos via intraperitoneal injection. Each group were intravenous or intraperitoneal injection of 0.5 mL PBS, or 0.5 mL N-DSC-exos (100 µg). The injections were given 3 days prior mating and then daily after conception until the fifth day of gestation, totaling six administrations. Male and female mice were mated 2:1 and have been observed at 8:00 and 14:00 every day since their mating. The day when vaginal plug appeared was identified as gestation day 1. This animal experiments received approval from the Human Research Ethics Committee of Renji Hospital ( Ethical Number: 2022-0056).

### Observation of embryo resorption rate

Mouse were killed by neck dislocation on day 12–14, following which their uteri were excised. We then documented the total number of implantations and resorption sites (signs of abortion) was recorded, in accordance with methodologies delineated in our prior studies [27, 28]. Resorption rate = [(number of total abortion)/(number of total implantation)] × 100%.

### Statistical analysis

Each experiment was replicated thrice to ensure consistency. The statistical analysis were performed with SPSS 17.0 software. Results are presented as mean ± standard deviation (SD), and graphs of the experimental data were generated with Graph-Pad Prism software. Two-tailed unpaired Student’s t-test was used to analyze the significance of differences between two groups, and one-way ANOVA was used for the comparisons of three or more groups. Comparison among multiple groups was carried out by one-way ANOVA followed by Tukey’s post hoc test. *p*-value < 0.05 was considered to be statistically significant.

## Results

### Identification of DSCs/ESCs by immunofluorescence

**Fig. 1:**
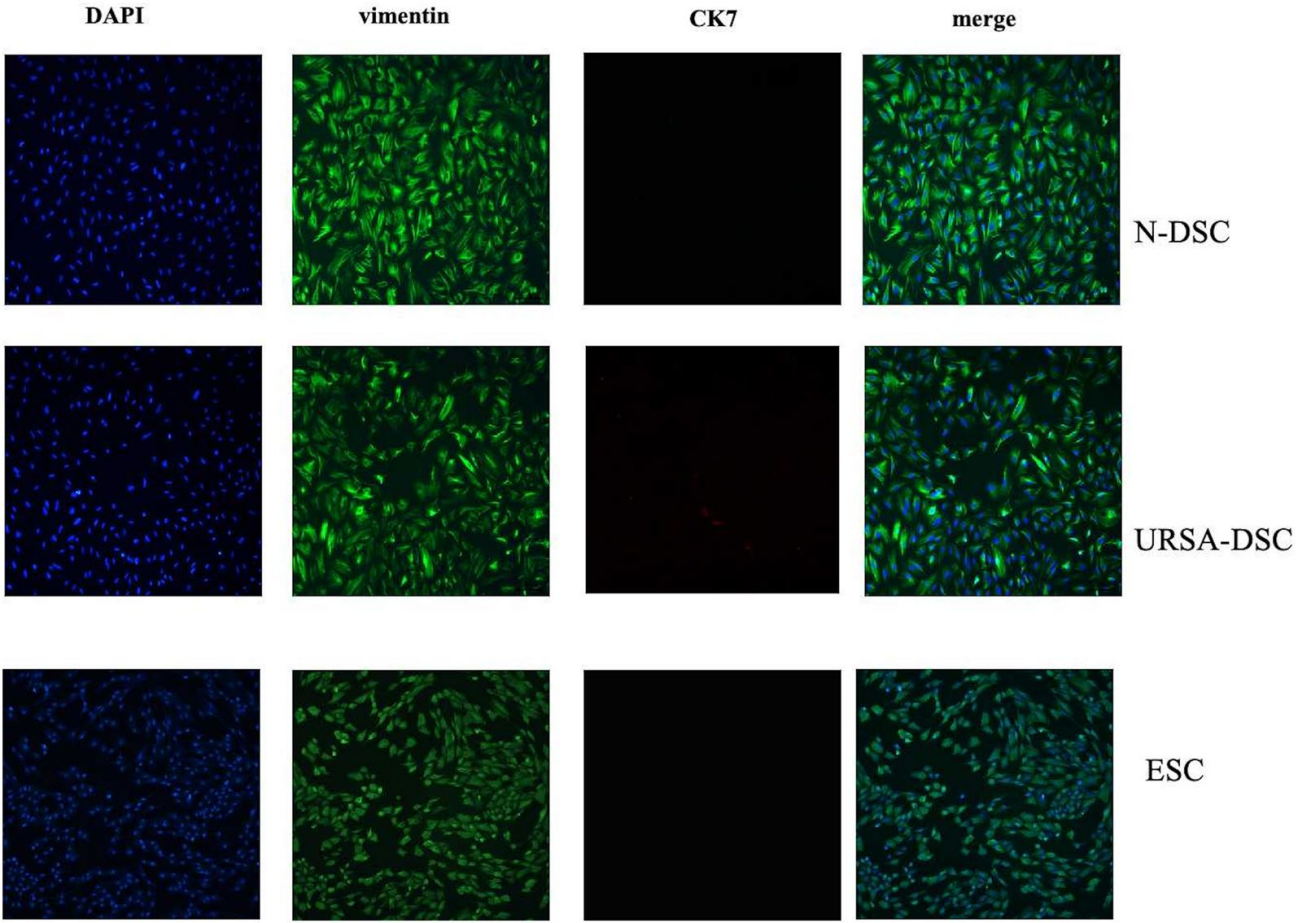
Identification of DSC/ESCs by immunofluorescence. Primary ESCs and DSCs were irregular polygonal. Moreover, compared with ESCs, DSCs showed a larger cell size with an abundant cytoplasm under an optical microscope, which was consistent with the literature reports, and they were identified as stromal cells based on their cell-specific expression determined via immunofluorescence staining: vimentin positive and cytokeratin 7 negative

Enzymatic digestion and the adherence technique have proven effective for isolating primary DSCs and ESCs, thereby significantly reducing fibroblast contamination. Primary ESCs derived from the secretory endometrium, and DSCs, obtained from the decidua in the early stages of pregnancy, exhibit an irregular polygonal. Notably, compared with ESCs, DSCs are distinguished from ESCs by their larger cell size with an abundant cytoplasm under an optical microscope, which was consistent with the literature reports. The phenotypic identification of these cells as stromal in origin was confirmed through immunofluorescence staining: vimentin (mesenchyme origin-specific, green) positive and cytokeratin 7 (epithelial cell-specific, red) negative (Fig. 1).

### Identification of N-DSC-exos and URSA-DSC-exos and ESC-exos

**Fig. 2:**
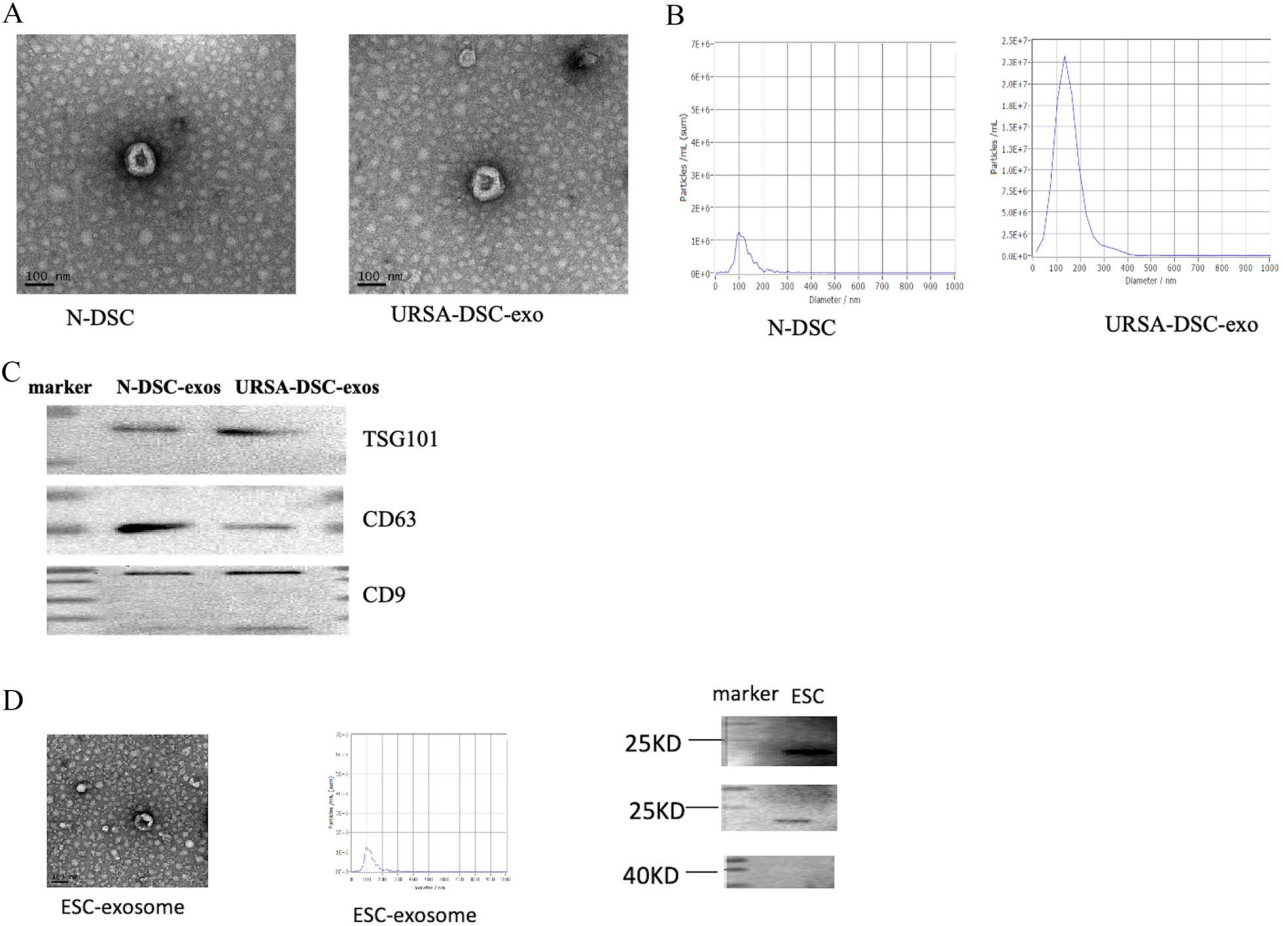
Exosome identification: TEM, NTA and WB. **A** The exosomes were examined by transmission electron microscopy as described in the supporting information methods; **B** The particle sizes and concentrations were confirmed to be exosomes using NTA; **C** WB test results showed that the expression of secrete marker proteins TSG101, CD9 and CD63 could be detected in the exosomes extracted from N-DSC and URSA-DSC. The ESC-exos expressed typical exosomal markers, including CD9, CD63. Full-length blots/gels are presented in Additional file 2 named WB-original picture

We conducted an isolation and characterization of exosomes from the decidua of URSA patients and normal pregnancy. TEM image showed that the double concave disc-shaped bilayer membrane structure was observed in the N-DSC-exos, URSA-DSC-exos, and ESC-exos, similar to that of saucer, as shown in Fig. 2A. NTA displayed that the N-DSC-exos, URSA-DSC-exos and ESC-exos were rounded particles ranning from 30–200 nm. Specifically, N-DSC-exos had a particle concentration of 2.2 × 10^7^ particles/mL after a 300-fold dilution, leading to an estimated original concentration of 6.6 × 10^9^ particles/mL. The mode particle size measured was 161.3 nm, constituting 100% of the recorded peak area. The mean particle diameter was 164.6 nm. Likewise, URSA-DSC-exos exhibited a particle concentration of 9.2 × 10^7^ particles/mL with a 100-fold dilution, inferring an original concentration of 9.2 × 10^9^ particles/mL. The mode particle size for these was 146.2 nm, with the peak area at 99.9%, and a mean particle size of 150.8 nm, as depicted in Fig. 2B. For ESC-exos, the particle concentration was 1.8 × 10^7^ particles/mL after a 40-fold dilution, indicating an original concentration of 7.2 × 10^8^ particles/mL. The peak size for these exosomes was 103.1 nm, with the peak area covering 90.9% of the spectrum and an average particle size of 128.8 nm, as shown in Fig. 2D. The N-DSC-exos and URSA-DSC-exos expressed typical exosomal markers, including TSG101 and CD9, CD63 (Fig. 2C). The ESC-exos expressed typical exosomal markers, including CD9, CD63 (Fig. 2D).

### Tracing of exosomes

**Fig. 3:**
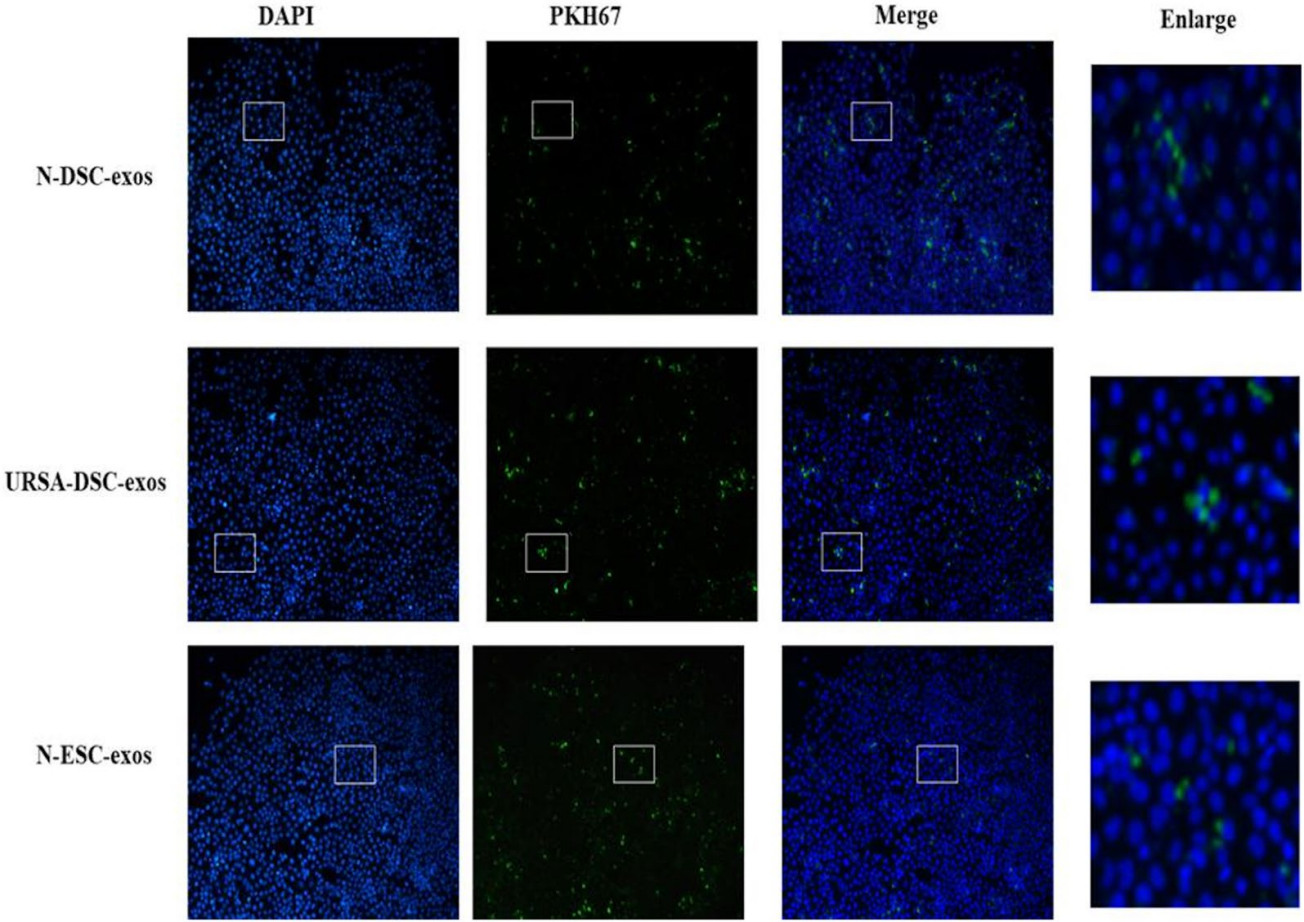
Trace of exosomes from decidual stromal cells. Laser confocal microscope displayed that green fluorescence was observed in HTR8/SVeno cells after co-cultured with PKH67-labeled DSC-exos/ESC-exos, indicating their efficient uptake of N-DSC-exos and URSA-DSC-exos and ESC-exos. (scale bar, 20 µm)

The PKH67 Green Fluorescent Cell Linker Kit (Sigma-Aldrich, USA) was used for the labeling of exosomes, as described in the Supporting Information Methods. HTR8/SVneo cell lines were co-cultured with PKH67-labeled N-DSC-exos or PKH67-labeled URSA-DSC-exos or ESC-exos for 0, 6, 12, 24, 48 h. Laser confocal microscope displayed that PKH67 signals were detected in the HTR8/SVneo, indicating their efficient uptake of N-DSC-exos and URSA-DSC-exos and ESC-exos (Fig. 3).

### DSC-exos increase the migration and invasion of trophoblast

**Fig. 4:**
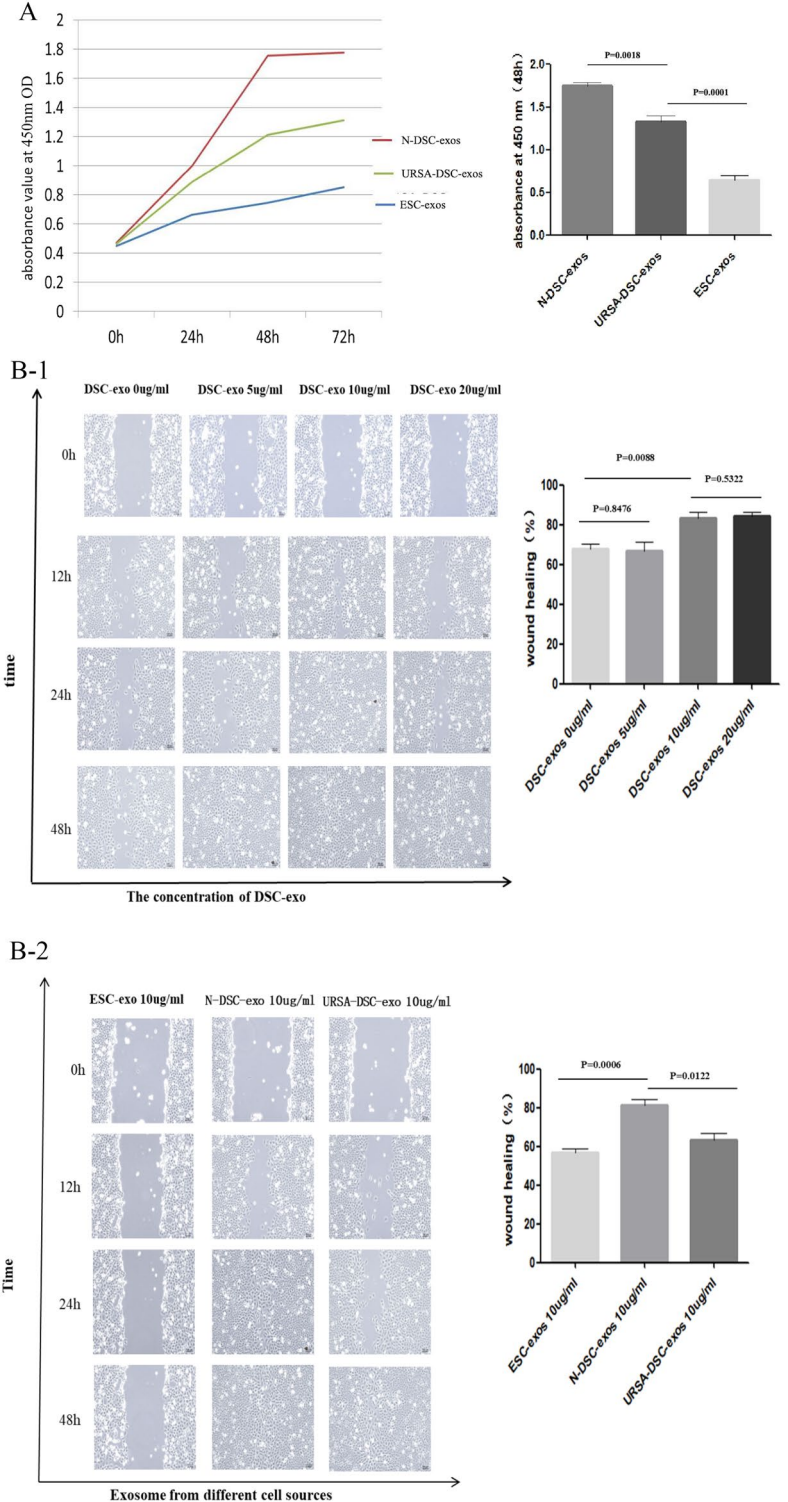

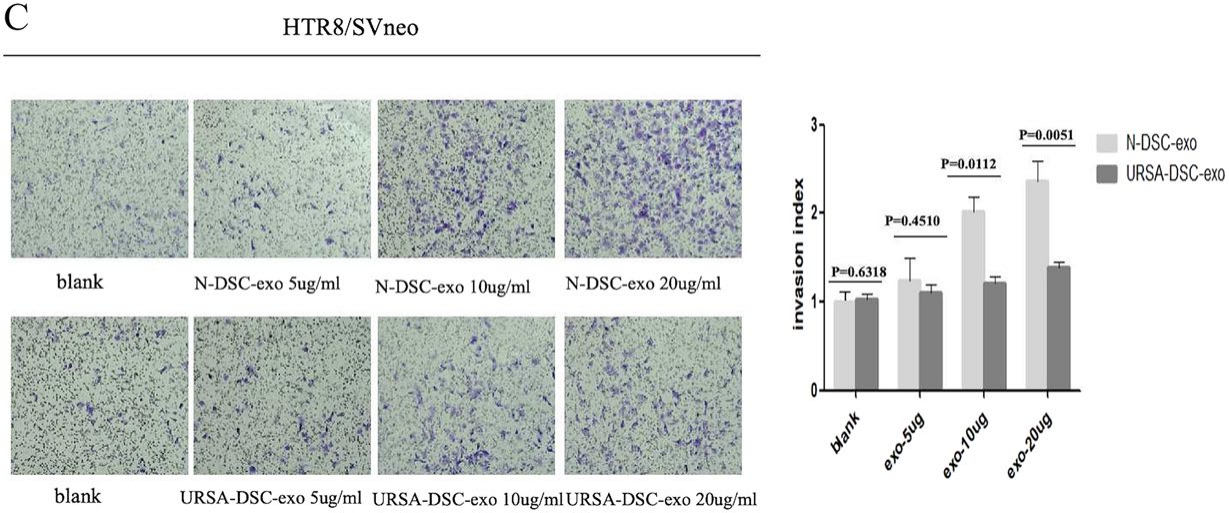
The effects of DSC-exos on the proliferation and invasion of trophoblast. **A** The influence of exosomes on HTR8/Svneo cell proliferation was assessed utilizing the CCK8 assay at a concentration of 20 µg/mL, which demonstrated that both N-DSC-exos and URSA-DSC-exos significantly enhanced cell viability compared to ESC-exos, with N-DSC-exos having a more potent effect. **B** Effects of different concentrations of exosomes on the migration ability in human trophoblast HTR-8/SVneo cells in vitro. The result revealed that N-DSC-exos markedly improved the migratory capacity of trophoblastic HTR8-SVneo cells. While ESC-exos and URSA-DSC-exos also elevated trophoblastic migration, their impact was substantially less than that of N-DSC-exos, with the most pronounced migratory response observed at an exosome concentration of 10 µg/mL. **C** Effects of different concentrations of exosomes on the invasiveness in human trophoblast HTR-8/SVneo cells in vitro. Transwell assays indicated that N-DSC-exos augmented the invasive potential of HTR8/SVneo cells to a greater extent than URSA-DSC-exos, and the maximal invasiveness was noted at an exosome concentration of 20 µg/mL. (magnification, × 20). Error bars, SD

To investigate the differential effects of exosomes on HTR8/SVneo cells, we introduced N-DSC-exos, URSA-DSC-exos, and ESC-exos into the culture medium. Cell proliferation was quantified using CCK8 assays. The results revealed that N-DSC-exos (absorbance = 1.750 ± 0.037) and URSA-DSC-exos (absorbance = 1.33 ± 0.068) significantly promoted cell viability in comparison to ESC-exos (absorbance = 0.644 ± 0.050) (*p* = 0.0001), with N-DSC-exos exerting a stronger effect (Fig. 4A). Furthermore, in wound healing assays, HTR8/SVneo cells co-cultured with 10 µg/mL N-DSC-exos exhibited a wound closure area of (83.366 ± 3.001)%, compared to (67.844 ± 2.715)% in the control group (*p* = 0.0088) (Fig. 4B-1). At the same exosome concentration, the wound closure area for the N-DSC-exos and URSA-DSC-exos groups were (81.196 ± 3.037)% and (63.250 ± 3.478)%, respectively, indicating a significantly enhanced migratory response in the ESC-exos group (56.735 ± 2.228)% (*p* = 0.0006) (Fig. 4B-2). This suggests that N-DSC-exos markedly improve the migration capability of trophoblast HTR8/SVneo cells, particularly at a concentration of 10 µg/mL. While ESC-exos and URSA-DSC-exos also facilitated trophoblast migration, their effects were considerably weaker than those of N-DSC-exos.

Invasion assays further demonstrated that with increasing concentrations of N-DSC-exos, the invasion index of trophoblasts were 1.289 ± 0.248, 2.025 ± 0.154, and 2.369 ± 0.223, respectively. In contrast, trophoblasts co-cultured with URSA-DSC-exos showed invasion index of 1.029 ± 0.058, 1.089 ± 0.073, 1.216 ± 0.071, and 1.388 ± 0.065 at escalating concentrations, which significantly less than the N-DSC-exos group (*p* < 0.05) (Fig. 4C). These findings from the transwell assays indicate that N-DSC-exos significantly enhance the invasive potential of HTR8/SVneo cells more than URSA-DSC-exos, with peak invasiveness observed at an exosome concentration of 20 µg/mL. Collectively, these results underscore the superior proliferative and invasive capabilities conferred by N-DSC-exos.

### DSC-exos affects the expression of EMT markers on trophoblast

**Fig. 5:**
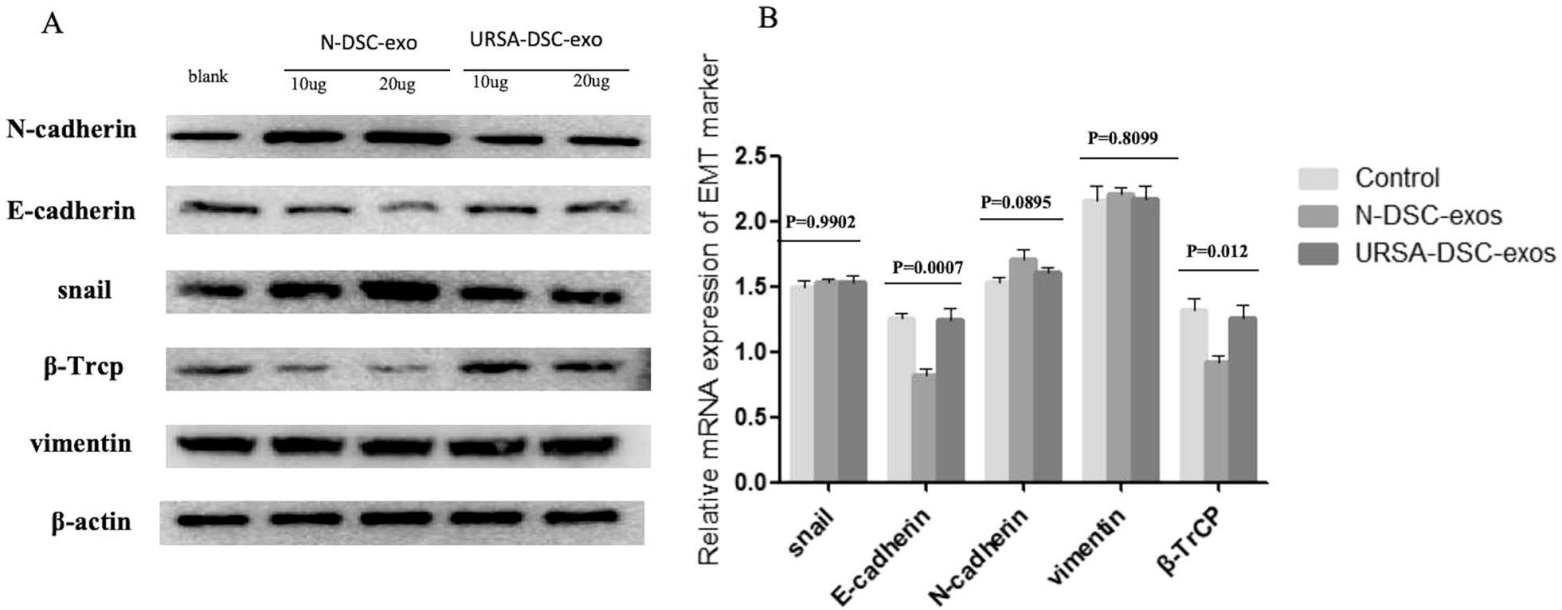
DSC-exos affects the expression of EMT markers on trophoblast. **A** Representative western blot images showing the relative levels of β-TrCP, E-cadherin, N-cadherin, vimentin, and snail proteins in HTR8/SVneo treated with N-DSC-exos and URSA-DSC-exos. **B** Quantitative real-time PCR and were performed to analyze the levels of β-TrCP, E-cadherin, N-cadherin, vimentin, and snail mRNA in HTR8/SVneo treated with N-DSC-exos and URSA-DSC-exos. Full-length blots/gels are presented in Additional file 2 named WB-original picture. Statistical analysis was performed using one-way ANOVA, Tukey’s post hoc test

**Fig. 6:**
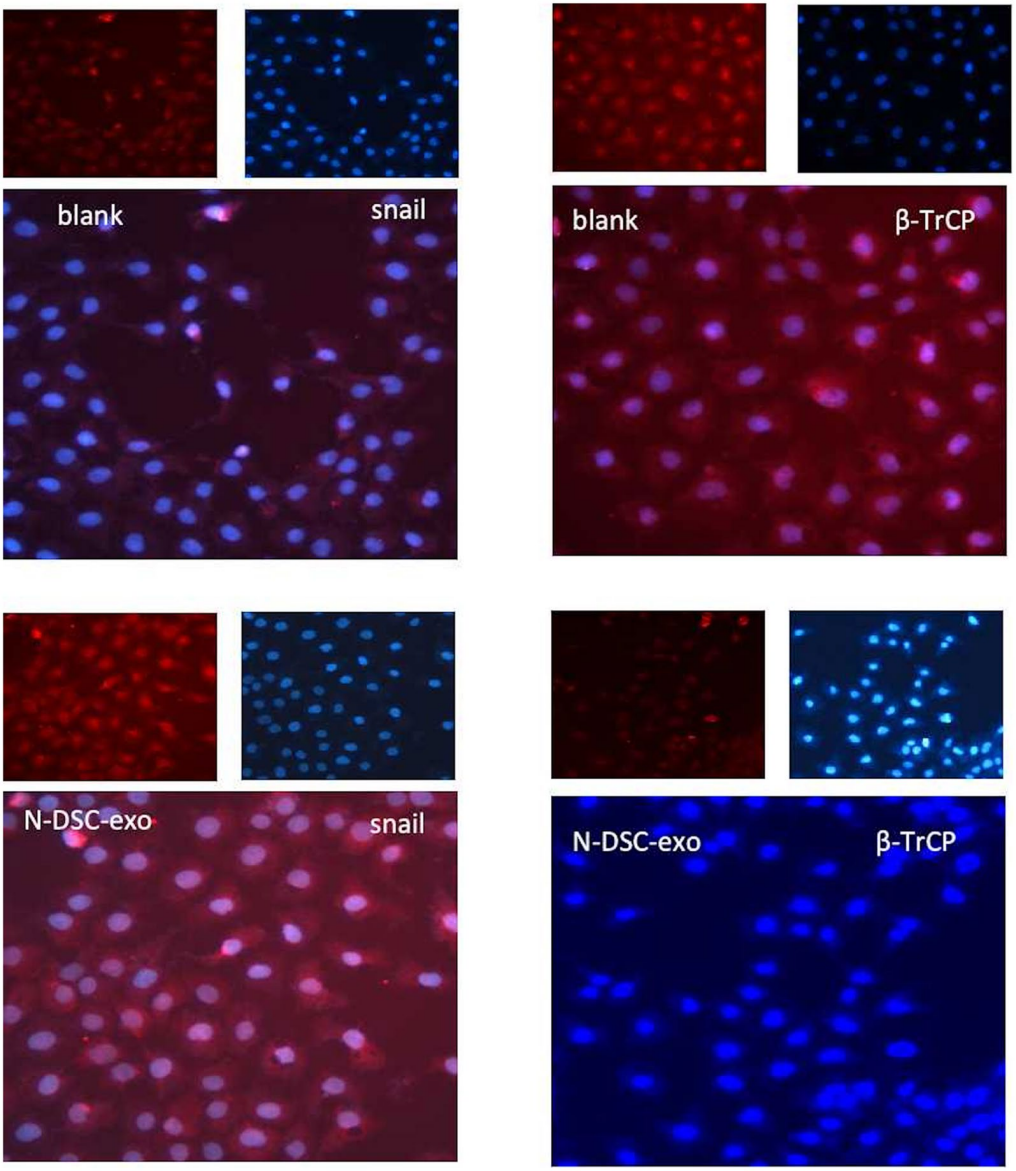
Immunofluorescence assay showing the subcellular localization of β-TrCP and snail. Immunofluorescence assays showed that N-DSC-exos led to a upregulation of sanil protein and down-regulation of β-TrCP in HTR8/SVneo cells

To assess the impact of DSC-exos on the regulation of EMT markers in trophoblast, quantitative real-time PCR and a subsequent WB analysis of EMT biomarkers were implemented. Quantitative PCR analysis revealed that the mRNA expression level of β-TrCP in HTR8/SVneo cells treated with N-DSC-exos was 0.917 ± 0.055. This was significantly lower compared to the control group (1.327 ± 0.086) and the group treated with URSA-DSC-exos (1.259 ± 0.090) (*p* = 0.012). Furthermore, E-cadherin mRNA levels were significantly reduced in HTR8/SVneo cells treated with N-DSC-exos (relative mRNA = 0.821 ± 0.047) in comparison to control group (relative mRNA = 1.261 ± 0.037) or those treated with URSA-DSC-exos (relative mRNA = 1.245 ± 0.088) (*p* = 0.0007). It is noteworthy that the mRNA expression levels of snail in N-DSC-exos, URSA-DSC-exos, and ESC-exos did not exhibit a significant differential expression. The relative mRNA expression levels were quantified as 1.490 ± 0.050, 1.530 ± 0.025, and 1.539 ± 0.046, respectively (*p* = 0.9902). Similarly, the expression levels of E-cadherin and vimentin did not exhibit any significant changes upon treatment with either N-DSC-exos or URSA-DSC-exos (Fig. 5B). WB revealed that the DSC-exos induced the expression of EMT biomarkers including snail, and N-cadherin, and inhibited the expression of E-cadherin and β-TrCP. The WB analysis further demonstrated that N-DSC-exos prompted an upregulation of snail and N-cadherin, while concurrently downregulating E-cadherin and β-TrCP (Fig. 5A). Additionally, immunofluorescence assay was employed to visualize the subcellular localization of β-TrCP and snail (Fig. 6).

### β-Trcp reduced snail expression in trophoblast cells via the ubiquitin–proteasome pathway

**Fig. 7:**
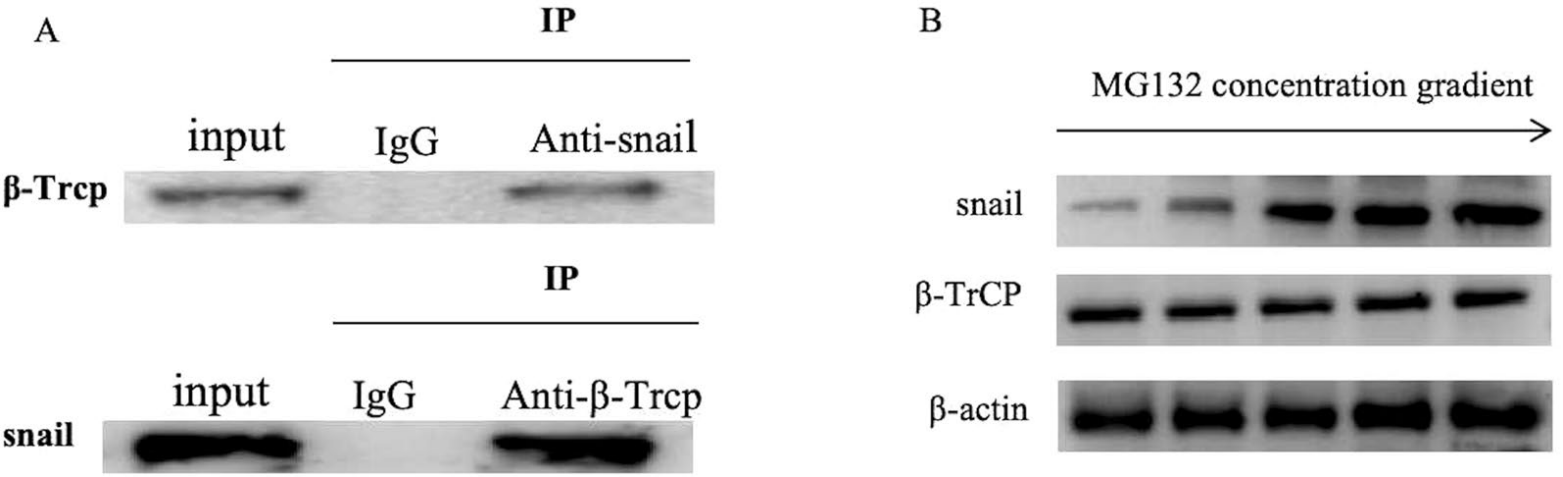
Verify the interaction of β-TrCP and snail. **A** Immunocoprecipitation suggested that β-TrCP interacts with snail: the WB results of the input showed that the target protein was expressed in the HTR8/SVneo cells, and β-TrCP can precipitate snail, meanwhile, snail can precipitate β-TrCP. **B** Changes in the level of snail and β-TrCP on the cell surface after treatment with the proteasome inhibitor MG132 (blank, 0.125 μM, 0.25 μM, 0.5 μM, 1.0 μM).The results showed that with the increase of MG132 concentration, the expression of snail gradually increased in a concentration-dependent manner, while the expression of β- TrCP had no significant change. Full-length blots/gels are presented in Additional file 2 named WB-original picture

Snail, a key factor in promoting EMT, has been reported to cause targeted degradation via the β-TrCP-mediated ubiquitination–proteasome pathway. Co-immunoprecipitation data indicate an interaction between snail and β-TrCP (Fig. 7A), suggesting β-TrCP’s role in snail’s regulatory diminishment. To confirm whether the degradation of snail by β-TrCP is dependent on the ubiquitination–proteasome pathway, HTR-8/SVneo were treated with the proteasome inhibitor MG132. Results showed that inhibition of the ubiquitination–proteasome pathway markedly enhanced the expression of snail on HTR-8/SVneo cells (Fig. 7-B).

### Down-regulation of snail reversed the effects of DSC-exos on trophoblast cell

**Fig. 8:**
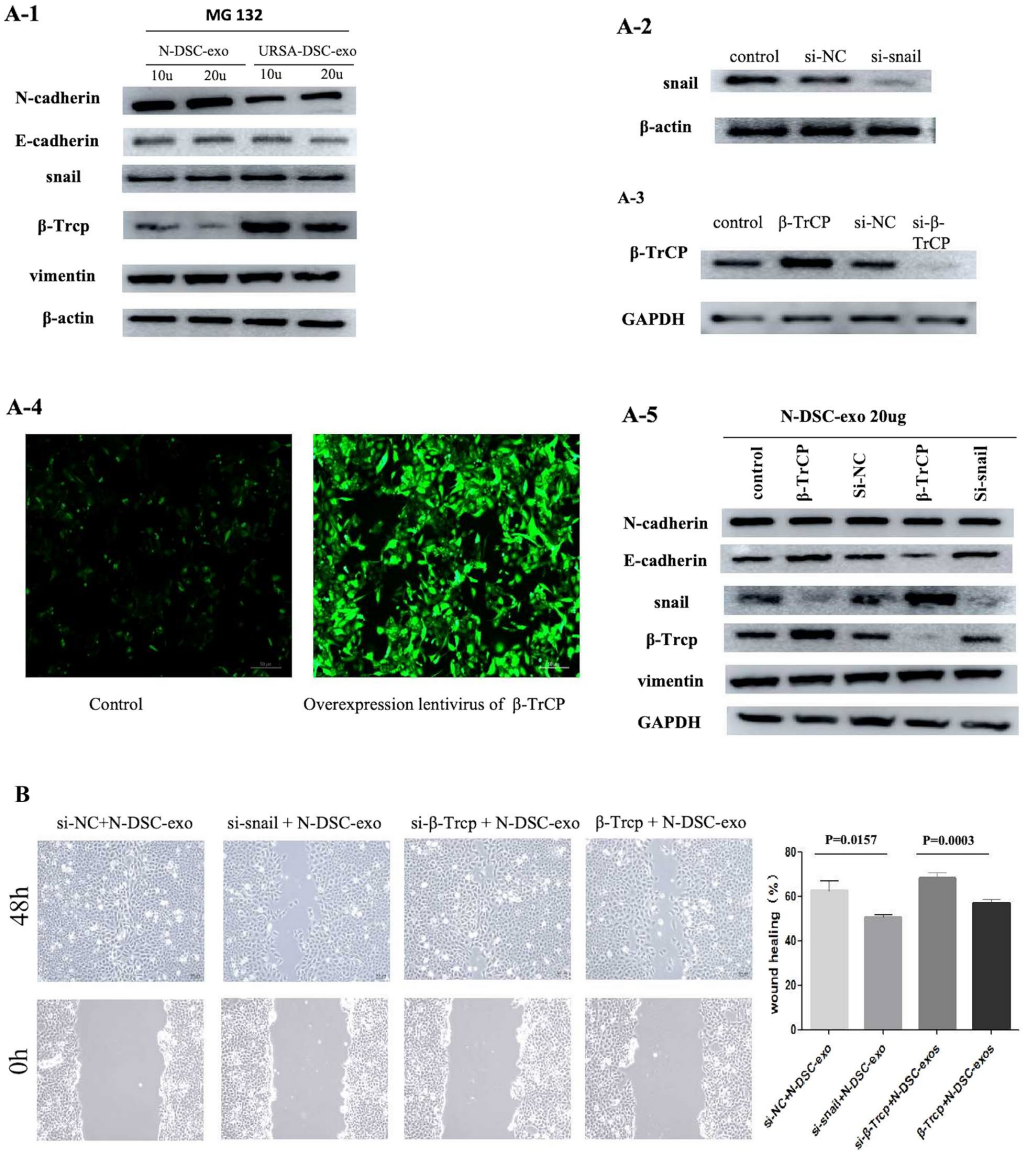
Down-regulation of snail or overexpression of β-Trcp reversed the effects of DSC-exos on trophoblast cell. **A-1** Representative western blot images showing the relative levels of β-Trcp, E-cadherin, N-cadherin, vimentin, and snail proteins in HTR8/SVneo treated with N-DSC-Exos + MG132 and URSA-DSC-exos + MG132. **A2-4** Representative western blot images showing the relative levels of β-Trcp, E-cadherin, N-cadherin, vimentin, and snail proteins in HTR8/SVneo transfected with si-snail, si-β-TrCP or infected overexpression Lentivirus of β-TrCP. Fig. 4 Fluorescence photos of infection with lentivirus. **B** Wound-healing assays were performed using HTR8/SVneo cells

To determine whether DSC-exos increasing the proliferation and invasion of trophoblast through the β-TrCP-mediated ubiquitination–proteasome pathway, trophoblast HTR-8/SVneo cells were treated with MG132, or transfected with si-snail or si-β-TrCP or infected β-TrCP overexpression Lentivirus. However, we found that down-regulation of snail did not affect β-TrCP expression. Furthermore, we found that MG132 blocking β-TrCP-mediated degradation of snail reversed the effects of DSC-exos on expression of EMT-related markers in trophoblast HTR8/SVneo cells including the snail, E-cadherin (Fig. 8A1). We next performed rescue experiments to investigate whether DSC-exos promoted trophoblast cell migration by inhibiting β-TrCP via upregulation of snail. HTR8/SVneo were transfected with si-snail, si-β-TrCP or infected overexpression Lentivirus of β-TrCP and WB verified the effect of knockdown or overexpression (Fig. 8A2-4).

**Fig. 9:**
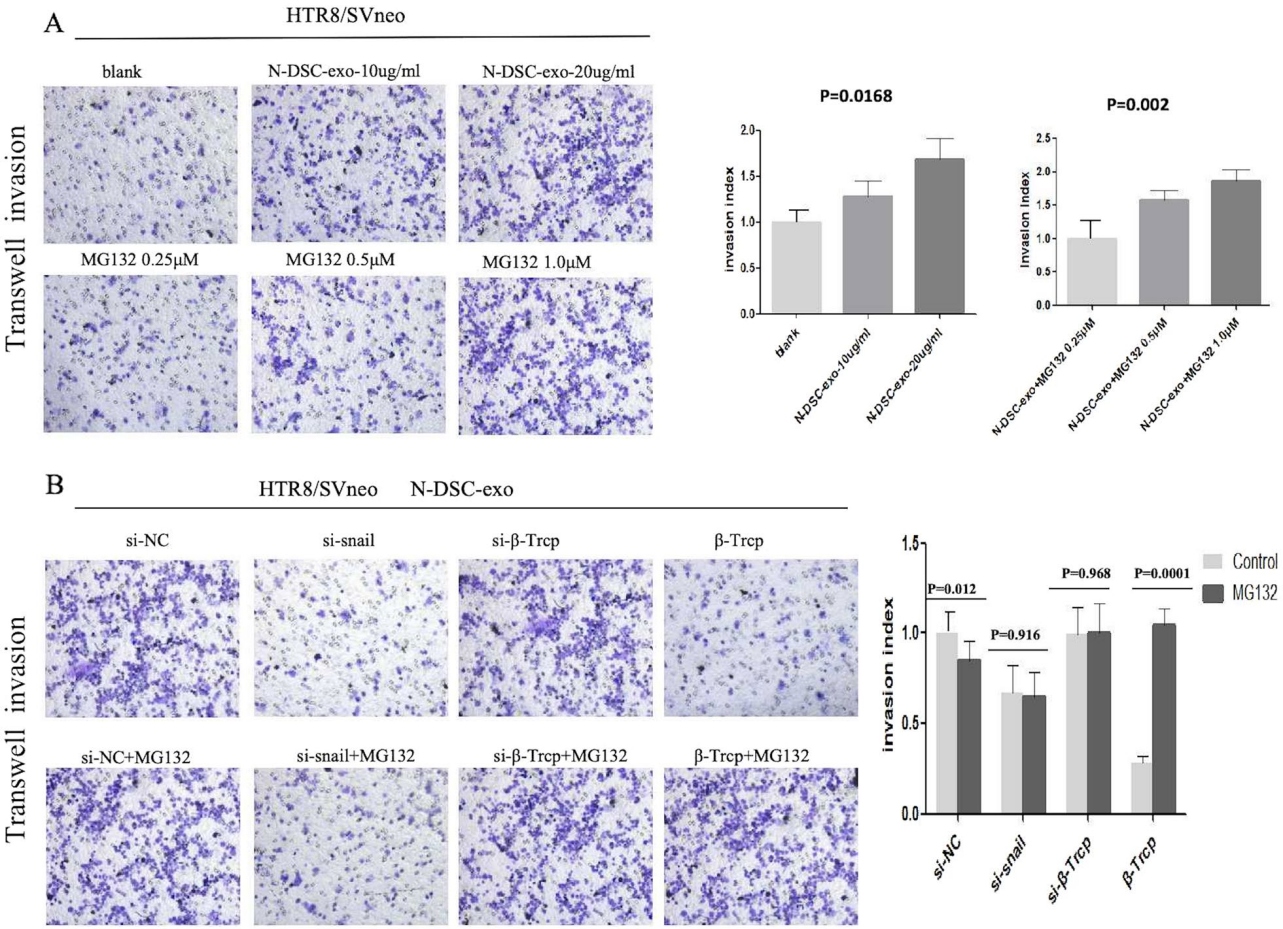
Down-regulation of snail or overexpression of β-Trcp reversed the effects of DSC-exos on trophoblast cell. **A**, **B** Transwell assays were performed to measure the invasion ability of HTR8/SVneo cells (magnification, × 20). Full-length blots/gels are presented in Additional file 2 named WB-original picture. Statistical analysis was performed using one-way ANOVA, Tukey’s post hoc test

Upon transfection with siRNA targeting snail (si-snail), the wound closure area in trophoblast cells co-cultured with N-DSC-exos was significantly reduced from (62.61 ± 4.46)% to (50.68 ± 1.35)% (*p* = 0.0157). In contrast, transfection with siRNA targeting β-TrCP (si-β-TrCP) resulted in a wound closure area of (68.42 ± 2.34)%, compared to (57.24 ± 1.42)% observed with β-TrCP overexpression (*p* = 0.0003) (Fig. 8B). Additionally, an increase in the concentration of N-DSC-exos correlated with an enhanced invasion index of the trophoblast cells, rising from 1.28 ± 0.17 to 1.69 ± 0.21 (*p* = 0.0168). The invasion index further escalated with increasing concentrations of the proteasome inhibitor MG132, with invasion index of 1.58 ± 0.14 and 1.86 ± 0.17 (*p* = 0.002) recorded (Fig. 9A). Conversely, trophoblast cells transfected with si-snail exhibited a decreased invasion index of 0.67 ± 0.16 compared with si-NC group (*p* = 0.0032). The invasion index for the si-β-TrCP group was 0.99 ± 0.15, while β-TrCP overexpression led to a significantly lower index of 0.28 ± 0.04 (*p* = 0.0003). The invasive capacity of trophoblasts was further increased from 0.28 ± 0.04 to 1.05 ± 0.09 (*p* = 0.0001) following the addition of MG132 to the β-TrCP overexpression group (Fig. 9B). Data from wound healing assays and Transwell assays showed that snail knockdown significantly reversed the DSC-exos induced elevated cell migration and invasion ability of HTR8/SVneo. Similarly, the increased invasiveness of cells caused by β-TrCP knockdown was significantly attenuated by down-regulation of snail in HTR8/SVneo cells. These results indicated that snail might be a downstream functional factor involved in β-TrCP regulation of trophoblast cell migration, invasion, and EMT process and DSC-exos may affect EMT process through the β-TrCP/snail ubiquitination–proteasome pathway.

### Elevated β-Trcp expression in villi from URSA patients

**Fig. 10:**
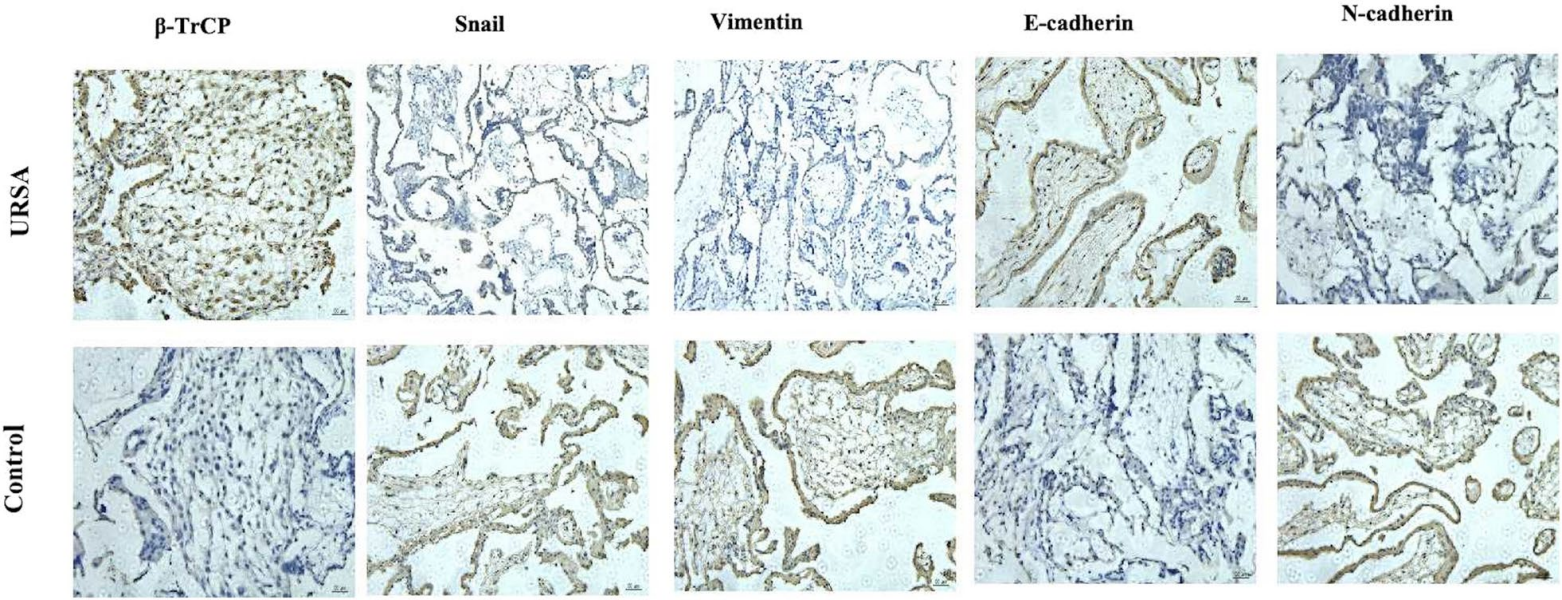
Immunohistochemistry showed the expression of EMT-related molecules. Representative images showing β-TrCP, snail, E-cadherin, N-cadherin, and vimentin protein expression in villi as detected by immunohistochemistry (magnification, × 20)

To investigate the potential role of β-TrCP and snail in the pathogenesis of URSA, samples of villi tissue were collected from URSA patients and healthy pregnancy women. Our qRT-PCR analysis revealed that the levels of β-TrCP mRNA expression in villi from URSA patients were significantly higher than the control group. Consistent with that finding, immunohistochemistry staining showed that the levels of β-TrCP protein in villi from URSA patients were much higher than those in villi from the control group. Conversely, the levels of snail in URSA tissues were much lower than those in the control tissues. Moreover, our results also showed that E-cadherin protein levels were elevated in the URSA group, while the levels of N-cadherin and vimentin proteins were reduced in the URSA group (Fig. 10 and Table 2).

### DSC-exos reduced the embryo resorption rate in vivo

**Fig. 11:**
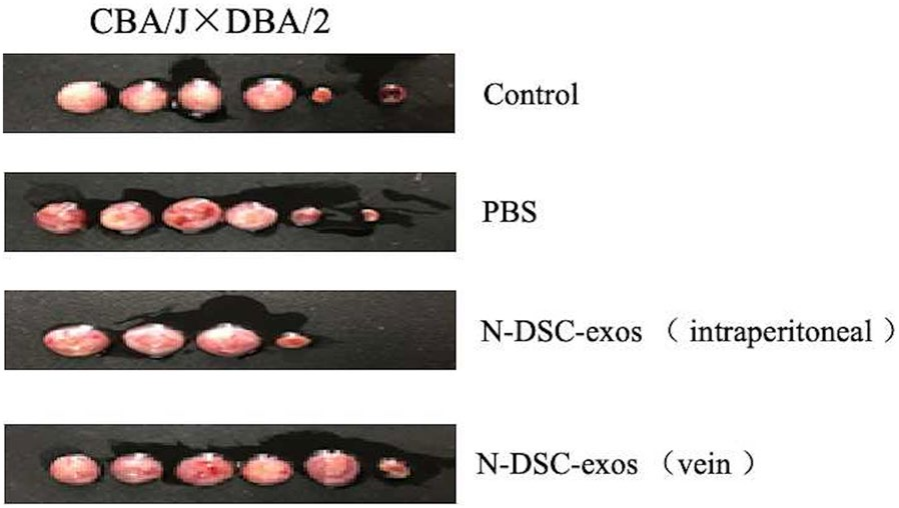
Representative images of embryo in the uterus from abortion mouse model treated with N-DSC-exos. Data depict the embryo resorption rate in pregnant mice treated with N-DSC-exos was significantly lower compared with the PBS-treated mice

To further examine the effect of the DSC-exos in vivo, DSC-exo were injected into the spontaneous abortion model pregnant rats. The embryo resorption rate was counted on the 12–14th day of pregnancy. As shown in Table 1, the embryo resorption rate was statistically significantly decreased in group 5 (embryo resorption rate = 19.35%) and group 6 (embryo resorption rate = 21.43%) when they compared with group 3 (embryo resorption rate = 25.67%) and group 4 (embryo resorption rate = 24.14%), respectively (*p* < 0.05). Furthermore, the group5 showed a statistically significant decrease in embryo resorption rate when compared with group6 (*p* = 0.0490) (Fig. 11, Table 1).

**Table 1:** The resorption rate of experimental groups

Group	Number of mice	Number of total embryo	Number of total resorption	Resorption rate
Control (CBA/J × Balb/c)				
1. Without treatment 2. Treated with N-DSC-exos	11 5	48 26	9 5	18.75% 19.23%
Abortion (CBA/J × DBA/2)				
3. Without treatment	13	74	19	25.67%^a^
4. Treated with PBS	6	29	7	24.14%^b^
5. Vein injected with N-DSC-exos	5	31	6	19.35%^c^
6. Intraperitoneal injected with N-DSC-exos	4	28	6	21.43%^d^

^a^*p* < 0.05, *vs* control group; ^b^*p* > 0.05, *vs* group without treatment; ^c^*p* < 0.01, *vs* group without treatment; ^d^*p* < 0.01, *vs* treated with vein injected with N-DSC-exo

## Discussion

RSA has attracted much attention due to its maternal injury and emotional attack. A successful pregnancy is dependent on efficient implantation and placentation [10]. Emerging evidence demonstrates that insufficient migration and invasion of trophoblasts play critical roles in the pathogenesis of RSA. Studies have shown that exosomes participate in intercellular communication by delivering their contents to recipient cells. Trophoblast-derived exosomes from the human placenta, in particular, have garnered significant interest for their potential role in pregnancy. It has been shown that during implantation, embryo secrete exosome and other extracellular vesicles, which can interact with the mother’s and embryonic cells [29, 30]. Numerous studies have also linked exosome to placenta dysfunction and to the pathophysiology of pregnancy [31–34].

Although placental exosomes play an essential role in preventing fetal rejection and complicating pregnancy [31–34], however the origin of exosomes plays important role in embryo implantation be confined only to trophoblast, fetus, endometrium, maternal peripheral blood or amniotic fluid, the roles of decidual stromal cell derived exosomes is less well known. This study focused on the DSC-exos because of their widespread presence in maternal–fetal interface and their possible critical role in embryo implantation and differentiation. We addressed on the biogenesis of DSC-exos, as well as their isolation, characterization, functional analysis, and therapeutic potential for pregnancy-related. We have developed a protocol for isolating highly purified DSCs from the normal pregnancy and URSA patients by enzyme digestion, then the exosomes of DSC were separated by ultracentrifugation [10], our study revealed the first demonstration that N-DSC-exos increased the trophoblast HTR8-SVneo proliferation, invasion and migration in a dose-dependent manner. Contrastingly, URSA-DSC-exos displayed a negligible impact on trophoblast differentiation compared with N-DSC-exos. Nevertheless, it remains to be determined whether URSA-DSC-exos contribute to the etiology of associated disorders by altering the EMT in trophoblasts, or if they are a result of existing imbalances.

As a well-known feature of trophoblast, losing their epithelial phenotype and transition to mesenchymal phenotype, which allowing them to invade and migrate into decidual of maternal [5, 35, 36]^.^ Trophoblast functions, particularly migration, invasion and proliferation are likely to be influenced by EMT regulators [5, 36]. EMT is one of the significant hallmarks of the maternal–fetal microenvironment and is involved in embryo implantation [5, 36, 37]. Thus, whether and how the DSC-exos in the maternal–fetus microenvironment promoting the EMT remain unclear. We validated the changes of EMT biomarker proteins including E-cadherin, N-cadherin, vimentin and snail by using N-DSC-exos, URSA-DSC-exos and ESC-exos co-cultured with trophoblast lines HTR8/SVneo, we found that exposure to N-DSC-exos resulted in a marked reduction in E-cadherin expression and a concomitant increase in N-cadherin and snail, with these changes demonstrating a dose-dependent response, however, although there was a decrease of vimentin protein in the villi of URSA patients, it was not directly linked to DSC-exos. Further research shows that although URSA-DSC-exos can modulated EMT biomarkers, their impact was significantly less pronounced than that of N-DSC-exos. Abnormal trophoblast invasion may reflect failure of trophoblasts to acquire phenotypic changes required for the mesenchymal migratory/invasive phenotype. E-cadherin, one of the more extensively studied adhesion molecules, is a key phonotypic marker of cells in the epithelial state [37], and reduced E-cadherin expression is associated with trophoblast acquiring a migration or invasion potential [5, 37]. Previous studies have shown that snail inhibits the expression of E-cadherin, snail is a well-known Zn-finger transcription factor that promotes EMT by promoting EMT-related biomarker expression [38–41]. N-cadherin is another member of the cadherin transmembrane glycoprotein family, which plays an important role in the formation of EMT, N-cadherin promotes the down-regulation of E-cadherin and cellular adhesion [41, 42], such cadherin switching is now considered a feature of EMT, supporting the transition from an epithelial to an invasive phenotype [40, 42–45]. It is becoming increasingly clear that DSC-exos may regulate key pathways that trigger EMT by modulating EMT biomarker protein levels. Nonetheless, the EMT encompasses a complex set of processes, and the precise mechanisms by which DSC-exos influence EMT, particularly how they induce a decrease in E-cadherin and an increase in snail and N-cadherin expression, are topics that merit further investigation.

Current knowledge of EMT in the human placenta is not comprehensive enough in the difference, expression and functional roles of a number of the well-known EMT biomarkers. To explore the mechanism of the effect of DSC-exos on EMT, in our investigation, we concentrated on how DSC-exos affect EMT transcription factors that are crucial for EMT signaling pathways. The transcriptional repressor snail, a member of the snail family of zinc-finger proteins, plays an essential role in regulating of invasion during embryo implantation and considered to be a potent inducer of EMT [40, 45, 46]. Localized predominantly in extravillous trophoblasts (EVTs) within the maternal decidua and to a lesser extent in the villous trophoblast cells, snail's expression suggests it plays a central role in trophoblast invasion [47, 48]. Consistent with this research report, our study showed decreased snail, N-cadherin and vimentin expression in the DSC of URSA by immunohistochemical, which may reflect inappropriate trophoblast proliferation, or impaired trophoblast differentiation, either of which could have adverse consequences. The zinc finger transcription factor, snail, functions as a potent repressor of E-cadherin expression that can induce an EMT, was found to be affected at the protein level by DSC-exos, rather than at the mRNA level in our study. Research demonstrated that snail displays β-catenin-like canonical motifs that support its β-TrCP directed ubiquitination, GSK3β-dependent phosphorylation, and proteasomal degradation [49–52]. Beta-transducin repeats-containing proteins (β-TrCP) is a substrate recognition subunit of the SCFβ − Trcp E3 ubiquitin ligase in the ubiquitin proteasome pathway [53, 54]. By regulating substrate degradation, β-Trcp participates in various cellular processes, including cell migration and invasion [55]. Research findings [55] that β-Trcp mRNA and protein expression were upregulated in preeclampsia placentas, and β-Trcp suppressed trophoblast invasion by inactivating snail via the ubiquitin proteasome pathway. Another study suggests that that miR-135a-5p promotes the migration and invasion of trophoblast cells by targeting β-TrCP [56]. In our research, we verified the interaction of snail and β-Trcp in trophoblast by co-immunoprecipitation assay, the result displayed that snail protein could be captured by β-Trcp and β-Trcp protein could be captured by snail in the trophoblast HTR-8/SVneo. Moreover, protease inhibitor MG132 treatment increased the expression of snail in a concentration-dependent manner, which confirmed that there was an interaction between β-Trcp and snail in the maternal–fetal interface. It is clear that β-TrCP regulates snail via ubiquitination and degradation, which may exert important physiological effects in trophoblasts.

Our further study showed that N-DSC-exos significantly inhibited the expression of β-TrCP, which caused an increase levels of snail, then down-regulation of E-cadherin, seemingly no effect on N-cadherin levels. Moreover, protease inhibitor MG132 or siRNA-snail reversed the effects of DSC-exos on the trophoblast, including the ability of proliferation, invasion, migration and the expression of EMT biomarkers, and a reduction degradation rate of snail protein when compared to a control group, which was consistent with previous literature reports that showed that β-Trcp plays an critical role in EMT by regulating the migration and invasion of trophoblast [55, 56]. To further examine the effects of the DSC-exos on the EMT of trophoblast in vivo, N-DSC-exos were injected into the spontaneous abortion model pregnant mouse. The results suggest that tail vein injection of DSC-exos significantly reduced the embryo resorption rate of pregnant mouse in spontaneous abortion model. We speculated that N-DSC-exos might mediate the biological behavior of trophoblast by suppressing of β-Trcp-mediated ubiquitination and degradation of EMT transcription factor snail, considering the prospects for using DSC-exos to therapy pregnancy pathologies, for example URSA. However, the question of DSC-exos intrinsic components and their possible biological functions remains open. Further studies are needed to determine their mechanism of DSC-exos on affecting the expression levels of pathway associated signaling molecules and downstream effectors, or by signal enhancing function interaction with EMT-associated nuclear factors.

## Conclusions

The N-DSC-exos mediated the biological behavior of trophoblast through regulation the degradation of EMT biomarker snail depended on β-TrCP. The URSA-DSC-exos caused insufficient migration and invasion of trophoblast because of disturbing of β-TrCP-mediated ubiquitination and degradation of EMT transcription factor snail, reversing this disorder will illuminate a novel mechanism in DSC regulation of trophoblasts and their role in URSA.

As shown in Table 1, the embryo resorption rate was statistically significantly decreased in group 5 (embryo resorption rate = 19.35%) and group 6 (embryo resorption rate = 21.43%) when they compared with group 3 (embryo resorption rate = 25.67%) and group 4 (embryo resorption rate = 24.14%), respectively. Furthermore, the abortion model group5 showed a statistically significant decrease in embryo resorption rate when compared with group6.

As exhibited in Table 2, the expression of N-cadherin, snail, and vimentin was markedly elevated in the villi of healthy early pregnant women when contrasted with those diagnosed with URSA. Concurrently, a significant decline in E-cadherin and β-TrCP expression was observed.

**Table 2:** Immunohistochemical staining score of β-TrCP and EMT-related molecules in villus

Category	Normal pregnant women (immunohistochemical staining score)	URSA patients (immunohistochemical staining score)
E-cadherin	1.000 ± 0.186^a^	2.286 ± 0.578^b^
N-cadherin	2.636 ± 0.419^c^	1.429 ± 0.291^d^
Snail	2.909 ± 0.4208^e^	1.657 ± 0.248^f^
Vimentin	2.636 ± 0.3636g	1.714 ± 0.221h
β-TrCP	2.455 ± 0.359^i^	4.143 ± 0.490^j^

^a,b^E-Cadherin, normal pregnancy group vs URSA group, *p* = 0.0174

^c,d^N-cadherin, normal pregnancy group vs URSA group, *p* = 0.0435

^e,f^Snail, normal pregnancy group vs URSA group, *p* < 0.01

^g,h^Vimentin, normal pregnancy group vs URSA group, *p* < 0.01

^i,j^β-TrCP, normal pregnancy group vs URSA group, *p* < 0.001

### Supplementary Information

**Additional file 1.** Characteristics of included study population.

**Additional file 2.** Original picture including Full-length blots/gels.

## Data Availability

The datasets used and analyzed during the current study are available from the corresponding author on reasonable request.

## Abbreviations

URSA: Unexplained recurrent spontaneous abortion
DSC: Decidual stromal cell
EMT: Epithelial–mesenchymal transition
TEM: Transmission electron microscopy
NTA: Nanoparticle tracking analysis
PE: Preeclampsia
FGR: Fetal growth restriction
RIF-EV: Recurrent implantation failure-extracellular vesicles
FER-EVs: Fertile women-extracellular vesicles
DSC-exos: Decidual stromal cell derived exosomes
URSA-DSC-exos: DSC-exos from URSA patients
N-DSC-exos: DSC-exos from normal pregnant women
ESC-exos: Endometrial stromal cells-derived exosomes
NP: Normal pregnancy
ESC: Endometrium
WB: Western blot
PVDF: Polyvinylidene fluoride
Co-IP: Co-immunoprecipitation
BSA: Bovine serum albumin
