# Supplementary material for: Decidual stromal cells-derived exosomes incurred insufficient migration and invasion of trophoblast by disturbing of β-TrCP-mediated snail ubiquitination and degradation in unexplained recurrent spontaneous abortion
Source: Eur J Med Res. 2024 Jan 9;29:39. doi: 10.1186/s40001-023-01598-2 (PMC10775448; doi:10.1186/s40001-023-01598-2)
Supplement: Supplementary file 1 — Additional file 1. Characteristics of included study population. [file 40001_2023_1598_MOESM1_ESM.docx]

**Supplenmentary Table 1 Characteristics of included study population**

|  |
| --- |

**Normal control(n=22) URSA(n=14)**

| Maternal age (y) 30.08±3.83 30.94±3.97  Gestational week（w） 7.99±0.71 8.06±0.87  BMI (kg/m^2^) 23.69 ±2.00 22.08±1.80  Number of pregnancies 2.54±0.97 3.18±0.98  Number of spontaneous abortions 0.375±0.49 3.062±0.93 |
| --- |
|  |
